# The role of digitalization and ESG on financial performance: An empirical analysis on the Energy and Utilities sectors

**DOI:** 10.1371/journal.pone.0314078

**Published:** 2025-02-27

**Authors:** Donato Morea, Gianpaolo Iazzolino, Carlo Giglio, Maria Elena Bruni, Giovanni Baldissarro, Elisa Farinelli

**Affiliations:** 1 Department of Mechanical, Chemical and Materials Engineering, University of Cagliari, Sardinia, Italy; 2 Department of Mechanical, Energy and Management Engineering, University of Calabria, Arcavacata, Italy; 3 School of Economics, Business and Accounting, University of São Paulo, São Paulo, Brazil; 4 School of Public Affairs, University of Science and Technology of China, Hefei, P. R. China; Universiti Teknologi MARA, MALAYSIA

## Abstract

In recent years authorities and regulators around the world are showing great interest in the concept of sustainability. Sustainable practices are a growing phenomenon around the world and there is increasing research on the correlation between Environmental, Social and Governance (ESG) and corporate financial performance (FP). In parallel with the increasing focus on ESG, digitalization has gained a pivotal role in the business environment. The paper wants to investigate the relationship between ESG factors and financial performance. Moreover, it tries to understand how digitalization influences that relationship. We use panel data regression using pooled ordinary least squares, fixed effects or least squares dummy variables. The panel covered by our study consists of a sample of listed companies belonging to the Energy and Utilities sectors observed from the year 2019 to 2021. In particular, our data set includes financial indicators closely related to the corporate profitability, sustainability indicators and an indicator use as a proxy of digitalization. The results provide interesting insights on how digitalization can moderate the relationship between ESG and profitability goals within the business environment, and especially the correlation that exists between sustainability and profit. The results suggest that ESG integration in corporate organizations positively affects FP because a strong ESG proposition enables businesses to grow both in existing and new markets. The findings further support the need to invest in digitalization since they show a substantial gain in financial efficiency, which can eventually boost corporate revenues. The research results support the minimization of the corporate social cost and, more generally, of social well-being. We contribute to the literature by studying the moderating role of investments in digital technologies in the context of sustainability, to understand whether or not digitalization can accelerate the impact of ESG on corporate profitability.

## 1. Introduction

Sustainability is now more than ever a business imperative. Many companies are struggling to deeply diving into the sustainability landscape, and they constantly try to face the sustainability challenges and business opportunities [1].

Sustainable practices are a growing phenomenon around the world and there is increasing research on the correlation between ESG (Environmental, Social and Governance) and corporate financial performance (FP) [2–5].

In particular, ESG factors have become extremely important because they allow a company’s ESG performance to be measured accurately and on the basis of standardized and shared parameters [4]. In 2019, ESG factors are recognized by the financial world as “key leverages” to achieve the goal of climate neutrality by 2050: the European “Green Deal”. ESGs reflect sustainability practices and can report a firm’s progress in implementing the Sustainable Development Goals (SDGs) [6]. Many studies in the literature show that sustainable investments, in addition to protecting the environment and society, offer an opportunity to achieve financial success [7]. In particular, it has been conducted a detailed study on both book and market performance, proving that they are positively impacted by ESG responsibilities [2] - see also the ESG risk-firm value relationship by [8]. Other researchers [9–12] proved that responsible behaviour improves firms’ performance, value and risk, under the framework of the stakeholder theory. However, contrasting results are reported in literature by another wave of researchers [5,13–18] that show how ESG and corporate financial performance are linked by a negative relationship, under the neoclassical theoretical framework. Hence, this proves that extant literature indicates two gaps consisting of inconclusive results on the ESG-performance relationship as well as the lack of relevant sector-specific studies [19–23].

In parallel with the increasing focus on ESG, digitalization has reshaped our world in profound and different ways, also revolutionizing the way companies do business. Digital transformation can reduce business costs and trade credit financing [24,25] and has positive effects on firm innovation and performance [26,27], despite some research [2] showed that digitalization alone is not able to impact significantly on corporate financial performance. The development of digital technology can reduce the transaction cost and the cost to obtain information [28]. With the application of digital technology in finance, corporate financing has been greatly facilitated [29]. Also, according to existing research [30,31] the sustainable development of businesses is positively impacted by digitalization. Digitalization opens to a more articulated investigation on the ESG-corporate financial performance relationship [22]. In fact, digitalization can play a key role in integrating ESG aspects within organizations, as digital technologies can be used to monitor and assess environmental impact, improve social practices, facilitate transparent and more effective governance [32], and promote corporate responsibility and fulfilment of social obligations [33]. On the other side, digitalization is found to have a significant positive impact on FP, that is further strengthened by ESG, in recent research on Korean listed firms [26]. However [26], conducted an in-depth analysis also on the different moderating role played by each ESG pillar, thus, revealing the intertwined and complex dynamics involving digitalization, single ESG pillars and FP. In this context, the development of digital finance can significantly improve corporate ESG performance, and especially environmental performance [34]. The adoption of ESG criteria and the integration of digital technologies can offer significant and synergistical opportunities for companies, enabling them to create long-term value, mitigate risks, and satisfy stakeholder expectations, thus, improving their overall performance [2,26]. Companies need to be aware of the environmental and social impacts of their activities and harness the potential of digital technologies to address the challenges and seize the opportunities offered by an increasingly sustainable and digital economy.

However, extant literature shows that digitalization poses a plethora of risks and challenges [35,36] related to distrust on the use of consumers’ data [37] as well as to its diffusion within developing countries [2].

Moreover, contrasting results are reported in literature not only about the digitalization-financial performance relationship, but also related to whether digitalization impacts the same way on firms located in developed or developing countries [2,37].

Hence, we identify and address the following gaps in literature: the hitherto under investigated link between ESG and FP, still missing a multi-country analysis and the focus on single ESG pillars; the role played by digitalization within the ESG-FP relationship.

Finally, we also identify the lack of a sufficient number of sectoral studies about the ESG-FP relationship and the role of digitalization. Beyond [38], proving the lack of significant ESG-FP relationships in Polish energy companies, and [39], that find positive effects of ESG on some performance indicators (i.e., return on equity (ROE), return on assets (ROA)) and negative on others (i.e., Tobin’s Q), the literature still misses a comprehensive understanding of the ongoing digitalization-ESG-FP dynamics in the Energy sector. Another contribution by [40] proves the very weak link among ESG and FP. Moreover, to the best of our knowledge, the Utilities sector is even less investigated, with [41] that warns about the potential reputational risks associated with the misalignment among ESG initiatives, performance and the quality of sustainability reporting. The need for creating value in the Energy and Utilities sectors via developing a good reputation among stakeholders and increasing innovation (including through digitalization) is also postulated by [42–44]. Overall, literature depicts a very fragmented and inconclusive view on the topic and shows the need to investigate it with sectoral studies considering their peculiarities. In fact, Energy and Utilities sectors are both intertwined to each other, and, above all, they are environmentally sensitive due to their impact on social and environmental issues [39,41]. Moreover, investigating and understanding such dynamics in the Energy and Utilities sector has not only a practical societal and environmental impact in the stakeholders’ view, but brings also a managerial interest to the firm, since those sectors are among the most connected to both the social and environmental challenges addressed by the ESG initiatives. Thus, investigating their efforts in terms of ESG and digital transformation, and how firms and the market react to such initiatives is key to contribute to the understanding of the digitalization-ESG-FP dynamics in the Energy and Utilities sectors.

The aim of the paper is twofold: first, we investigate the relationship between ESG factors and FP; second, we investigate the role of digitalization, and we try to understand how digitalization can influence that relationship, also discussing our findings against the inconclusive results in the literature. We contribute to the literature by studying the moderating role of investments in digital technologies in the context of sustainability, to understand whether digitalization can accelerate the impact of ESG on corporate profitability. In detail, we contribute by means of increasing the granularity of the statistical analysis compared to previous studies [2], since we focus not only on the overall ESG score, but also on each single pillar (i.e., E, S and G). This is both a methodological contribution and a literature one. As for the theory, we enrich the theoretical understanding of the investigated relationships under the lenses of the instrumental stakeholder theory (IST), also providing an extended version including a moderator variable like digitalization. Finally, our findings contribute to the ongoing discussion on the role of digitalization between ESG and corporate financial performance by showing that in a multi-country and multi-continent context there is no significant positive moderation of digitalization overall, whilst some specific pillars impact negatively on some financial variables. This result is derived mainly from developed countries (i.e., Europe, Oceania and America) and is in contrasts with the moderating role of digitalization in Indonesian firms [2].

The present paper draws from IST to address the following research questions:

*RQ*_*1*_ *: Is there a relationship between ESG and FP?*

*RQ*_*2*_ *: Is there a relationship between E, S, G and FP?*

*RQ*_*3*_ *: How does digitalization moderate the relationship between ESG and FP?*

The paper is organized as follows: Section 2 presents the literature review. Section 3 describes the research methodology and the data set used. Section 4 presents and discusses the empirical results. Finally, section 5 concludes the paper, highlighting the implications and limitations of the study as well as the future research directions.

## 2. Literature review

### 2.1. Extended instrumental stakeholder theory

IST belongs to the overall Stakeholder Theory, intended as an umbrella term covering different theoretical frameworks that deal with the understanding of firm-stakeholder relationships and their performance outcomes [45,46] categorize Stakeholder Theory into descriptive, normative and instrumental frameworks, whereas IST offers a particularly nuanced perspective on the relationship between stakeholder management and corporate performance. In fact, this theoretical approach conceptualizes firms as hybridized engines with a twofold mission. On the one side, they are conceived as mechanisms geared to wealth formation and the achievement of purely economic objectives; on the other side, firms are seen as actors with environmental responsibilities [47] as well as social and moral duties that connect closely business and society as a whole [2]. Such stakeholder relationships are managed based on ethical norms, mutual trust, and cooperation and aim at creating a sustainable competitive advantage [12,45]. In fact, frequent operating ways to pursue such objective are ESG disclosures that are geared to engage stakeholders, satisfy investors’ requirements, build trust [48] as well as to support the implementation of sustainability initiatives enhancing competitiveness and corporate FP [49,50].

Hence, IST is particularly useful in hybrid contexts characterized by both profit maximization and societal goals [44,51]. Under this perspective, the choice of the Energy and Utilities sectors for such an IST-based research setting is coherent with extant literature, since those sectors target firm- and societal-level objectives [42,43] confirm that research on ESG impacts is even more relevant to Utilities and Energy sectors, that are affected by reputational and natural resources issues [42].

Therefore, this is the typical application context of ESG investments and disclosures whereas FP are intertwined with social and environmental impacts. Furthermore, it has been chosen to apply the IST to investigate the impact of ESG on FP in Indonesian firms, including context factors (i.e., digitalization) [2,45,52,53].

However, the positive impact of IST-based stakeholder relationships on firm performance has no repercussions on the (limited) application of IST on the managerial/firm side [45,54,55] explains this contradiction by stating that little theoretical efforts have been made to understand how IST-based stakeholder relationships could lead to sustainable competitive advantage and increased performance. Moreover, this lack of understanding is complemented by the underexplored downsides of IST-based stakeholder relationships, operating represented by detrimental independent variables that could reduce FP [56,57]. Also, context-related factors, operating represented by moderator variables referred to the changing environments (i.e., faster pace of innovation, knowledge- and technology-intensiveness), need to be integrated in IST-based research [52,53], thus, representing a way to extend the original version of the IST [12,45]. Finally, the three limitations to IST applications further justify the theoretical framework adopted by this study.

### 2.2. Hypotheses development

The strand of scientific research evaluating the impact of ESG factors on a company’s FP has been enriched in recent years with a multiplicity of studies and empirical models [14,58,59], including also context-related factors coherent with the IST [45,52,53]. However, there is still little clarity regarding the link between ESG and FP, as empirical results are not univocal, possibly due also to the hybrid nature of the ESG-FP relationship that involve social and environmental impacts to be investigated under the lenses of the IST [47,49]. Some studies report a positive relationship among ESG and FP [9–11,60], whilst others report negative results [5,13–18]. The differences among those studies are manifold. The country contexts of those studies as well as the variables considered differ from each other, thus, depicting a multi-faceted and unresolved discourse literature [19–23]. As such, empirical studies could be divided into two groups: studies that do find a positive/negative correlation [61,62], and studies that do not find a correlation between ESG factors and financial indicators [23,38,63–65]. Specifically [61], showed that financial factors (e.g., cash flow) have a positive effect on ESG performance, while ESG performance has a positive effect to fundamental factors (e.g., sales revenue) [66]. found that ESG and all its three dimensions (environmental, social and governance) have a significant positive association with ROA, thus, confirming that firms have a double role aimed at achieving economic goals as well as social and environmental responsibilities, coherently with the IST [2,38,47] analyzing energy companies in Poland, finds no significant correlation between ESG factors and FP indices, as ROA and ROE [67]. showed that ESG score is positively associated with long-term firm value, but not with short-term profitability [39], analyzing the impact of ESG on performance indicators such as ROE, ROA, and Tobin’s Q in energy companies, finds positive correlation between ESG and ROE, but negative correlation with ROA and Tobin’s Q. Some studies show that a curvilinear relationship exists between ESG and FP [68] used Bloomberg’s ESG disclosure score as an indicator of a company’s ESG and concluded that the impact of ESG on finance had a U-shaped trend. Also [69], provided evidence of the inverted U-shaped trend of ESG impact on FP [70] found that the implementation of ESG practices has a convex U-shaped influence on profitability, meaning that firms will benefit when ESG investments exceed a certain level; in contrast, the nonlinear U shape occurs in the E and G components, but not in the S components of individual ESG initiatives. Similarly, the relationship between ESG and firm size, can also be explained by a curvilinear relationship [71] observed that firm size and sustainability reporting have an inverse U-shaped relationship, concluding that as firm size increases, the relative cost advantage induces firms to engage in more sustainable practices with the hope of gaining access to additional resources. In this context, previous studies have found that larger companies are more CSR (Corporate Social Responsibility) oriented than smaller companies because they are under constant scrutiny from their stakeholders [72,73]. This result is coherent with the IST, whereas stakeholder relationships are developed by paying attention to ethics, trust and cooperation [12,45].

In September 2015, the United Nations underlined the role of digital technologies in improving sustainability in the SDGs of the 2030 Agenda [74], which includes 17 goals and 169 specific projects. The SDGs are created to achieve a better sustainable future for all countries regarding social, ecological, and economic needs, as articulated by the UN in 2015 [75]. Nonetheless, there is little research currently available on how the SDGs affect firm performance [76], despite the extended IST framework recommends considering context-related variables, including those linked o technological dimensions like digitalization [45,52,53]. The SDGs’ goals are to create innovative, sustainable approaches and initiatives that will reduce environmental pollution while assisting in reducing poverty and creating jobs and community support [77], coherently with the IST that postulates that social and moral duties connect closely business and society as a whole [2]. SDGs have evolved into a shared objective, value, and a top priority of corporate organizations [78]. According to [79], a strong performance in achieving SDGs can have a positive impact on both product and financial markets. This can help companies gain market share and attract financial support. In recent years, research has paid special attention to digital transformation [80], as companies through digital transformation have begun to change traditional management strategies as well as governance approaches [32,81]. Enterprises use digital technologies to make a complete change of their products, services, processes, and models, thus, implying a complex organizational renewal and a more nuanced transformation of management practices [22,32,33]. This transformation enables them to build competitive advantage while generating ecological and social effects [34,82], thus, realizing the twofold mission – i.e., about firm competitiveness and societal/environmental impacts - of the IST [12,45,48,50]. The diffusion of digital technologies and culture is one of the key enablers to the achievement of the SDGs. Digital is, directly, one of the key goals related to SDG 9, to business, innovation, and infrastructure, but it also has indirect impacts on many other goals. The process by which digital transformation influences enterprise ESG performance has not received much attention in this context, preventing from reaching a shared consensus among field scholars [2,19–21,23,35–37]. To the best of our knowledge, only one study [2] have investigated the moderating role of digitalization on the ESG-FP relationship. However, the results are obtained by investigating mostly different variables (e.g., Tobin’s Q, cash flow, debt ratio) without an in-depth consideration of single ESG pillars (i.e., E, S and G). Moreover, the research has been conducted under different scenarios of analysis compared to our study [2]. First, the two works utilize different data sources: firms listed in the Indonesian Stock Exchange vs our Refinitiv Eikon dataset - that we selected (also) because it is specialized in the provision of ESG scores. Second, the research efforts are conducted within different macro- and micro-economic contexts: only Indonesian firms (i.e., single-country data) relying on a developing economy context vs firms included in Refinitiv Eikon from countries of the American/European/Oceanian continents (i.e., mostly developed economies). Thus, our study ensures a wider (multi-country) geographical and macro-economic coverage. Third, our work weighs less the pre-pandemic period as we focus on 2019–2021, while the other research [2] focus on 2017–2021, whereas 60% of their firm-year data refer to an obsolete time by now. This further enforces our explanation that the recent investments in digitalization have not shown all their effects, yet, and more time is needed to capture their impact. Fourth, variables are partly different either in terms of conceptualization or operationalization. Fifth, the granularity adopted for the ESG scores is higher in our study, as we do not only consider the overall ESG score, but also the single scores for each pillar: E scores, S scores, G scores. Sixth, we make a specific sectoral choice for our analysis (i.e., energy and utilities sectors), thus, our results offer a higher reliability and managerial impact on those sectors, since they are more immediately applicable in corporate contexts.

Other scholars have focused their efforts limitedly on one-to-one relationships involving digitalization, ESG or FP, without deepening the three variables as a whole [83]. found that digitalization can significantly improve corporate ESG scores, in particular digitalization can help reduce information asymmetries between managers and employees, external investors, and managers, improving corporate reputation and increasing enterprise governance (G) scores [32,33,84]. This result suggests that the theoretical lenses of the IST represent a valuable framework to contribute to the understanding of how the mutual trust and the reputational assets are affected by ESG and digitalization within the context of IST-based stakeholder relationship management [48]. The application of digital technologies can be an effective way to address environmental problems, such as air pollution, carbon emissions and climate change [34,85,86] studied the impact of Open Innovation (OI) on firm value by integrating ESG factors, finding that OI improved firm value, but this was through the mediation of ESG factors. The impact of digital transformation on ESG performance is less pronounced when there is increased environmental uncertainty, as seen from an external viewpoint. However, from an internal standpoint, larger-scale enterprises experience a more significant positive effect of digital transformation on ESG performance [87]. As for specific technologies like Industry 4.0, their integration with circular economy (CE) practices improves organizational performance and has a positive impact on companies’ financial performance [75]. Moreover [88], examine the impact of corporate transformation towards Industry 4.0 on FP in a set of manufacturing firms located in the United Kingdom. Findings suggest that such a transformation has accelerated and brought positive effects on FP. They also proved a moderating effect of ESG in such a relationship. In another study [89], investigated rooted in resource-based theory (RBT), natural-resource-based view, and dynamic capabilities in order to respond to the call made by [90] aimed at exploring “how business strategies that improve environmental performance continue to evolve with agility and creativity to maintain sustainable competitive advantage” [89].

Finally [91], articulates how governance and sustainability play a key role in companies’ efforts to implement corporate social responsibility practices, to achieve climate change and sustainable performance coherently with the United Nations 2030 agenda [75]. Moreover, this work depicts how the current debate on ESG research and digital technologies remains open to different perspectives, contexts and settings, and identifies future avenues of research linked to the exploration of each ESG pillar such as: environmental [92–94], social [95], and governance [96–101].

Hence, given the contradicting findings and the underexplored and unresolved issues linked to the ESG-FP relationship, the role of each ESG pillar, and the impact of digitalization as a moderating variable, we identify two main gaps in literature that deserve further research efforts. The first gap is about the underexplored nexus between ESG and FP, not covering multiple geographical and macro-economic contexts neither the role played by single ESG pillars. The second gap is about the unresolved question of whether (and how) digitalization moderates the ESG-FP relationship.

Despite literature offers examples of either positive and negative impacts of ESG on FP as well as of the moderating role of digitalization, the overall tendency is in favour of hypothesizing a positive influence of ESG and digitalization on the investigated relationships. Then, we formulate the following hypotheses:

*H1: ESG has a significant positive impact on FP;*

*H2: Each ESG pillar (E, S and G) has a significant positive impact on FP;*

*H3: Digitalization has a significant positive impact on the relationship between ESG and FP.*

## 3. Materials and methods

In this section, the empirical research is described. We illustrate the sample and data used, the main variables and the methodology.

### 3.1. Sample and data

The panel covered by our study consists of a sample of listed companies belonging to the Energy and Utilities sectors observed from the year 2019 to 2021. We chose the Energy sector because it is an environmentally sensitive sector and, in addition, companies belonging to this sector are under intense social and environmental monitoring. Moreover, other empirical studies, have shown a weak link between ESG scores in the Energy sector and returns [40]. The Utilities sector is also very sensitive to ESG factors because there could be potential misalignments between performance of ESG factors and quality of sustainability reporting, and this is particularly relevant for the Utilities sector because of the existence of reputational risks associated with their activities [41]. In particular, the sample consists of 149 companies belonging to the Utilities sector and 154 belonging to the Energy sector from around the world. Our data set includes financial indicators closely related to the corporate profitability, as ROA, ROE, Revenues and Earnings before interests, taxes, depreciation, and amortization (EBITDA) margin, sustainability-based indicators, the ESG scores and the three pillars (E, S, G) considered separately. In addition, we use as a proxy of digitalization, an indicator that measures whether the company supports the SDG 9 Industry, Innovation, and Infrastructure. The financial, sustainability and digitalization data are collected from Refinitiv Eikon ASSET 4, a database widely used in the financial industry [40], and one of the most reliable and accurate sources of ESG information [102].

The Appendix includes the table with part of the data considered (Table 1.A).

**Table 1:** Descriptive statistics and pairwise correlations (source: authors’ elaboration).

*Descriptives*										
*Variable*	**Obs**		**Mean**		**Std. Dev.**		**Min**		**Max**	
**ESG score**	909		55.48		17.927		7.028		92.811	
**E score**	909		53.829		23.258		1.055		96.345	
**S score**	909		55.405		22.067		2.161		96.499	
**G score**	909		58.148		21.735		4.583		98.563	
**EBITDA MARGIN**	909		0.106		3.266		−71.435		1.243	
**REVENUES**	909		22.111		1.645		12.086		26.661	
**ROE**	909		0.083		0.159		−1.256		1.173	
**ROA**	909		0.029		0.053		0-.305		0.337	
**Size**	909		21.421		1.428		16.419		25.453	
**Leverage**	909		1.408		3.434		0		96.14	
***Pairwise correlations***										
***Variable***	**(1)**	**(2)**	**(3)**	**(4)**	**(5)**	**(6)**	**(7)**	**(8)**	**(9)**	**(10)**
**(1) ESGscore**	1.000									
**(2) Escore**	0.883*	1.000								
	(0.000)									
**(3) Sscore**	0.881*	0.714*	1.000							
	(0.000)	(0.000)								
**(4) Gscore**	0.542*	0.219*	0.278*	1.000						
	(0.000)	(0.000)	(0.000)							
**(5) EBITDA MARGIN**	0.083*	0.086*	0.043	0.078*	1.000					
	(0.012)	(0.009)	(0.194)	(0.019)						
**(6) REVENUES**	0.426*	0.466*	0.332*	0.142*	0.315*	1.000				
	(0.000)	(0.000)	(0.000)	(0.000)	(0.000)					
**(7) ROE**	0.105*	0.085*	0.162*	−0.001	0.099*	0.148*	1.000			
	(0.002)	(0.010)	(0.000)	(0.975)	(0.003)	(0.000)				
**(8) ROA**	0.066*	0.065*	0.136*	−0.051	0.217*	0.170*	0.695*	1.000		
	(0.045)	(0.048)	(0.000)	(0.128)	(0.000)	(0.000)	(0.000)			
**(9) Size**	0.466*	0.516*	0.401*	0.086*	0.141*	0.545*	0.155*	0.117*	1.000	
	(0.000)	(0.000)	(0.000)	(0.009)	(0.000)	(0.000)	(0.000)	(0.000)		
**(10) Lev**	−0.032	0.014	−0.043	-0.053	0.025	−0.021	0.070*	−0.078*	0.008	1.000
	(0.339)	(0.682)	(0.196)	(0.110)	(0.457)	(0.536)	(0.034)	(0.018)	(0.806)	

*** p < 0.01, ** p < 0.05, * p < 0.1.

The results of the descriptive statistics are presented in Table 1.

### 3.2 . *Main Variables*

The dependent variables that we used are Revenues, EBITDA margin, ROE and ROA.

The variable Revenues is the total revenues from all of company’s operating activities, as defined on Refinitiv. Therefore, we decided to treat it as an indicator of operating performance, that is, a firm’s ability to generate value through core business activities. This variable was transformed to logarithmic form to avoid problems of heteroscedasticity among the residuals and improve model stability. As [103], we decided to use EBITDA margin as an indicator of FP. It is a useful indicator for measuring the firm’s operating profitability and is calculated by dividing EBITDA with Revenue. The higher EBITDA margin is, the higher firm’s profitability and it is useful for analysis between competitors because it is not affected by the different accounting methods between firms for depreciation expenses [104]. Companies’ profitability is often expressed by the ROA and ROE [69]. ROA is a very important indicator since it provides information on the ability of the enterprise to generate income relative to the value of its assets [105]. ROE measures a company’s FP and effectiveness in using shareholder equity, evaluating the profitability relative to shareholder’s equity [106].

The independent variables that we used are: ESG score, E score, S score, G score and SDG 9. In this study, the sustainability performance was measured using the ESG pillar score provided by the Thomson Reuters Eikon database. The scoring process involved over 450 measurements of corporate sustainability performance, assigning score values between 0 and 100. A higher score indicates better performance [107]. ESG score is calculated through a weighted average of the three pillars E, S, and G, also expressed on a scale of 1 to 100. In particular, the environmental pillar concerns a firm’s environmental responsibility. According to [108], it measures how well a company adopts the best policies and makes the most investments to minimize environmental risk and take advantage of environmental possibilities. The social pillar demonstrates a company’s dedication to the community, including areas such as health, safety, workplace diversity, training, labour rights, employee satisfaction, and customer satisfaction. On the other hand, the governance pillar concerns the implementation of effective corporate governance practices [109]. Finally, regarding the digitalization, we used a proxy that measures whether the company supports the SDG 9 Industry, Innovation, and Infrastructure. A dummy variable, SDG 9, was constructed: it is equal to 1 if company is supporting Goal 9 of SDG to build resilient infrastructure, promote sustainable industrialization and foster innovation, 0 otherwise.

### 3.3. Control variables

Referring to previous studies, we selected company size and financial leverage as control variables. Firm size (Size) is an essential control variable to be considered because the larger the company is, the more sensitive the enterprise may be to improve its FP. Therefore, for companies of significantly different sizes, their willingness to manage ESG performance and the economic results can be significantly different [1]. Financial leverage (Lev) provides insight into a company’s capital structure and was calculated as the ratio of total debt to total equity. In addition, we include country and sector dummy variables to avoid common endogeneity and control for both sector and country features.

The complete set of variables used in this study can be found in Table 2.

**Table 2:** Study variables (source: authors’ elaboration).

Variable type	Variable name	Variable code	Variable definition
Independent variables (IV)			
	ESG total score	ESGscore	Aggregate ESG score of the corporation as calculated by Refinitiv Eikon ESG database
	Environment Pillar Score	Escore	Environmental performance score of the corporation calculated by Refinitiv Eikon ESG database
	Social Pillar Score	Sscore	Social performance score of the corporation calculated by Refinitiv Eikon ESG database
	Governance Pillar Score	Gscore	Governance performance score of the corporation calculated by Refinitiv Eikon ESG database
	SDG 9	SDG9	Dummy variable which was assigned a value of 1 if company supporting SDG 9, 0 otherwise.
Dependent variables (DV)			
	EBITDA Margin	EBITDA_MARGIN	The ratio of EBITDA to Revenue
	Total Revenues	REVENUES	Total revenues from all of company’s operating activities
	Return on equity	ROE	Company’s FP and effectiveness in using shareholder equity, evaluating the profitability relative to shareholder’s equity.
	Return on Assets (FP)	ROA	Income after taxes/Total assets
Control variables (CV)			
	Size	Size	Natural log of the total assets of the corporation
	Leverage	Lev	Total Debt/Total Equity

### 3.4. Methodology

We use panel data regression using pooled ordinary least squares (OLS), fixed effects or least squares dummy variables (LSDV) and random effect models. Other studies used the same methods to study the relationship between ESG and FP [110,111]. To select an appropriate model, we initially performed a Lagrangian Multiplier (LM) test, which yielded a p-value of 0.000. This indicates that the random effect is more suitable than pooled regression. Subsequently, the F-test results reveal that the fixed effect is superior to pooled regression. Lastly, the Hausman test confirms that the fixed effect model is better than the random effect model. However, since the models include several time-invariant binary variables (e.g., country), in the standard panel data framework the estimation can be carried out using the LSDV with a fixed-effect approach [110]. The study incorporates ESG score, financial indicators, control variables, heterogeneity analysis, robustness and endogeneity tests, a large sample size, and latest data to enhance the rigor and credibility of its findings. It also incorporates control variables, conducts robustness and endogeneity tests, uses a large sample size, and utilizes sophisticated statistical software, such as STATA 17.0, for empirical analysis. To test the relation between ESG and FP, we used both the total ESG score (Models 1 to 8) and the individual pillars (Models 9 to 16) in the regression models. Second, to try to understand how digitization may affect this relationship, we test the moderating role of digitalization in the FP-ESG association where the interaction term between ESG and SDG 9 (the variable in the models 5 to 8 is called ESGs_SDG9_*it*_ =  ESG_*it*_ * SDG9_*it*_; in the models 13 to 17 is called Npillar_ SDG9_*it*_ =  Npillar_*it*_ * SDG9_*it*_) is included. From a methodological point of view the novelty of our work is the use of an indicator of digitalization, related to the SDG 9, as a moderator in the relationship between ESG and FP. The result of diagnostic tests for both models can be found in the Appendix (Tables 2.A, 3.A, 4.A and 5.A).

The models employed are as follows:

*FP*_*it*_ *= α*_*1*_ *+* β_1_ *ESGScore_*it*_ *+* β_2_ *SDG9_*it*_ *+* β_3_ *Size_*it*_ *+* β_4_*Lev_*it*_ *+* β_5_* ∑countryDummy *+* β_6_ * ∑SectorDummy *+ ∈*_*1it*_ (1, 2, 3, 4)
*FP*_*it*_ *= α*_*2*_ *+* β_7_ *ESGScore_*it*_ *+* β_8_ *SDG9_*it*_ *+* β_9_ *ESGs_SDG9_*it*_ *+* β_10_ *Size_*it*_ *+* β_11_*Lev_*it*_ *+* β_12_* ∑countryDummy *+* β_13_ * ∑SectorDummy *+ ∈*_*2it*_ (5, 6, 7, 8)
*FP*_*it*_ *= α*_*3*_ *+* β_8_*Escore_*it*_ *+* β_9_*Sscore_*it*_ *+* β_10_*Gscore_*it*_*+* β_11_*SDG9_*it*_ *+*β_12_*Size_*it*_ *+* β_13_*Lev_*it*_ *+* β_14_* ∑countryDummies *+*β_15_* ∑countrySector *+ ∈*_*3it*_ (9, 10, 11, 12)
*FP*_*it*_ *= α*_*4*_ *+* β_16_ *Escore_*it*_ *+* β_17_ *Sscore_*it*_ *+* β_18_*Gscore_*it*_*+* β_19_*SDG9_*it*_ *+* β_20_ *Epillar_SDG9_*it*_ *+* β_21_ *Spillar_SDG9_*it*_*+* β_22_ *Gpillar_SDG9_*it*_ *+* β_23_ *Size_*it*_ *+* β_24_*Lev_*it*_ *+* β_25_* ∑countryDummies *+* β_26_ * ∑countrySector *+ ∈*_*4it*_ (13, 14, 15, 16)

Where *FP*_*it*_ is the value of FP (Revenues, EBITDA margin, ROE or ROA) of the company I at the time t, whilst *α*_*1,*_ *α*_*2,*_ *α*_*3,*_ *α*_*4*_ are constant terms and *∈ *_*1it*_ *∈ *_*2it*_ *∈ *_*3it*_ *∈ *_*4it*_ are residual terms. In model 1-8, ESG represents the sustainability indicator, SDG 9 a proxy of digitalization, E_Dig the moderating variable, Lev is the leverage ratio (measured by total debt to total equity), Ln_Size represents firm size, measured by natural logarithm of the total assets of the last year available. In model 9-16, Escore represents the environmental pillar score, Sscore is the social pillar score, Gscore is the governance pillar score; Epillar_Dig, Spillar_Dig, Gpillar_Dig are the moderating factors. The description of the control variables is the same as model 1-8.

## 4. Results and discussion

Strong multicollinearity among the independent variables in the model can affect the reliability of the results of the mediation effect model analysis [1]. To test multicollinearity, statistical methods such as Pearson’s correlation test and variance inflation coefficient (VIF) test can be used. In our study, we used Pearson’s correlation test to evaluate all variables in the model, and the results are presented in Table 1. We find a high correlation coefficient between the ESG score and the three pillars. This result is normal because the ESG score is calculated on Refinitiv as a weighted average of the score of the three pillars. To avoid multicollinearity problems, we used the ESG score and the three pillar scores separately and proposed different models. The absolute value of the correlation coefficient among the remaining variables is 0.545 at most. Based on statistical theory, significant multicollinearity is typically considered to occur when the correlation coefficient between independent variables exceeds 0.8. As a result, it can be concluded that the model developed in this research does not exhibit any significant multicollinearity issues.

Table 3 presents the results of the regression models. We found that the only FP indicator on which ESG positively impacts are Revenues. This effect is statistically significant with a p-value < 0.01. This result shows that the positive unit change in ESG score allows for an estimated average increase in Revenues of 0.70% or 0.50%. This result is in line with the study by [112], where it is shown that ESG factors positively impact on corporate revenues. This is mainly because customers reward companies that adopt ESG strategies, increasing FP in the short term. This finding aligns with [113] study, which found a positive correlation between a company’s ESG scores and its revenues. The study suggests that implementing ESG policies can effectively attract consumers. As stated by [42], both the Utilities and Energy sectors do not have a very good reputation because of their environmental impact, but ESG investments do increase credibility and visibility with stakeholders, and this can positively impact revenue. Likewise, also [44] align with our findings, highlighting that Utilities sector has a hybrid objective that is both profit-oriented and aimed at creating value at the societal level [43]. confirm that ESG-related benefits for firms are even more relevant to sectors like Utilities and Energy, that rely strongly on both their reputational assets and natural resources [42]. We also find that ESG does not have a significant impact on other financial indicators. Regarding digitalization, we can see that it does not significantly moderate the relationship between the overall ESG score and FP.

**Table 3:** Results of the regression model (source: authors’ elaboration).

*Regression results of model 1–4*				
Variable	EBITDA MARGIN	REVENUES	ROA	ROE
**ESGscore**	0.00221706	.00500018 *	0.00012782	0.00058393
**SDG9**	0.11891379	−0.00827512	0.00045407	−0.00542476
**Lev**	0.01028243	−0.01193123	−0.00123983 *	0.00296892 *
**Size**	0.20808041 *	0.9746548***	0.00266789	0.01312905**
**Country FE**	YES	YES	YES	YES
**Industry FE**	YES	YES	YES	YES
**_cons**	−6.3176289***	0.7695537	−0.03707924	−0.22566056**
***Regression results of model 5–8 (with the moderating variable)***				
**Variable**	**EBITDA MARGIN**	**REVENUES**	**ROA**	**ROE**
**ESGscore**	0.01027382	0.00765352**	0.00014208	0.00072059
**SDG9**	1.2772722	0.37320775	0.0025041	0.01422232
**ESGs_SDG9**	−0.02026414	−0.0066736	−0.00003586	−0.0003437
**Lev**	0.00983031	−0.01208013	−0.00124063 *	0.00296125
**Size**	0.20683261 *	0.97424386***	0.00266569	0.01310789**
**Country FE**	YES	YES	YES	YES
**Industry FE**	YES	YES	YES	YES
_cons	−6.6983134***	.64418267	−.03775296	−.2321174**
***Regression results of model 8–12***				
**Variable**	**EBITDA MARGIN**	**REVENUES**	**ROA**	**ROE**
**Escore**	0.00195774	0.00627218***	−0.00029025 *	−0.00107717**
**Sscore**	−0.00834902	−0.00424263 *	0.00053246***	0.00178543***
**Gscore**	0.01374637 *	0.00381546**	−0.00009624	−0.00002149
**SDG9**	0.13559605	−0.00245377	−0.00069019	−0.00825099
**Lev**	0.01219871	−0.01307727	−0.00113172 *	0.00341532 *
**Size**	0.22189541 *	0.96900028***	0.00295645 *	0.01508361***
**Country FE**	YES	YES	YES	YES
**Industry FE**	YES	YES	YES	YES
**_cons**	−7.028188***	0.83088478	−0.04328701	−0.27358434**
***Regression results of model 5–8 (with moderating variable)***				
**Variable**	**EBITDA MARGIN**	**REVENUES**	**ROA**	**ROE**
**Escore**	0.01562699	0.00771154**	−0.00021331 *	−0.0012192**
**Sscore**	−0.02024359	−0.00522457 *	0.00043656**	0.00192153***
**Gscore**	0.02206246**	0.0068871***	−0.00005662	0.00027348
**SDG9**	1.6124061 *	0.47159338 *	0.00247928	0.02668997
**Epillar_SDG9**	−0.03141005 *	−0.00391387	−0.00016516	0.00023289
**Spillar_SDG9**	0.02349903	0.0020722	0.00019611	−0.00023721
**Gpillar_SDG9**	−0.01805948	−0.00637502 *	−0.00008769	−0.0005901
**Lev**	0.01073933	−0.01268691	−0.00113962 *	0.00352753 *
**Size**	0.23129674 *	0.97185463***	0.00301218 *	0.01532067***
**Country FE**	YES	YES	YES	YES
**Industry FE**	YES	YES	YES	YES
**_cons**	−7.766207***	0.57714683	−0.04565007	−0.29499465***

Legend: *  p < 0.05; ** p < 0.01; *** p < 0.001

*** p < 0.01, ** *p < 0.05,* *  *p < 0.1*

Moreover, Table 3 shows the results of the regression models whereas the relationship between each of the three ESG pillars and FP and digitalization was tested.

The results indicate that the E-score has a statistically significantly negative effect on ROA, showing that the expense of ESG practices becomes a cost to shareholders, limiting investment opportunities and overall performance [17]. Spending money on environmental goals (e.g., pollution control) increases expenditure, lowers profitability, and diminishes competitive advantage [114]. In addition, our results show that the S-pillar has a positive and highly significant (p-value <  0.01%) impact on ROA. This result is in line with the study of [115]. We can therefore state that to increase firm profitability, the management of the companies should pay particular attention to the social component of the ESG practices since the S score has the largest relevance in the association with ROA. The negative and significant association between the E pillar score and ROE suggests that companies with higher E pillar scores tend to have lower ROEs. This may be because companies that are more focused on environmental responsibility may be less focused on maximizing profits [106]. Furthermore, it’s possible that firms that place a higher priority on environmental responsibility are more inclined to invest in long-term initiatives that provide lower returns in the short term. In contrast to environmental pillar E, our results show that social pillar S has a positive and significant impact on ROE. Social practices and activities are considered as stimulating methods of corporate efficiency, and thus, are more successful in generating better FP [116]. The results confirm that investments in social activities, such as training, professional development programs and human rights policy, can improve business operational efficiency. In this context, our results suggest that social activities positively impact on FP, and this is consistent with the literature. In this field [117], showed that the social activities have a positive effect on FP in the long run [43]. stated that these benefits could be stronger in the sectors that depend more on reputation, brands, and large quantities of natural resources, such as the energy sector. The negative link between pillar E and these indicators of business efficiency, could be due to increased costs. In fact, the study of [118] states that implementing an environmental quality management strategy has associated different costs items. Additionally, adopting an environmental quality management policy may result in challenges related to shifting supply chain relationships and addressing moral dilemmas [119]. Regarding the relationship between ESG pillars and revenues, the results show that pillars E and G significantly and positively impact them, exceeding the negative and significant impact of the pillar S. The results obtained for the E and G pillars align with the findings of [113], which indicate that stronger ESG practices, particularly in the areas of environment (E) and governance (G), are linked to enhanced operating performance. This improvement can be attributed to the relationship with operating expenses, rather than asset turnover or increased revenues. Additionally, the positive impact of the governance component of ESG on financial performance demonstrates that investing in this pillar allows businesses to attract a diverse range of creditors and investors. Better governance serves as a market signal and attracts more customers, leading to an increase in revenue [112]. The positive relationship between ESG and corporate revenues may be related to the fact that, as supported by [51], companies in the Utilities sector have a hybrid nature in which both the logic of profit maximization and the goal of creating value for society coexist. Investment in ESG factors would certainly lead to the creation of value for the company and consequently an economic return, in this case in terms of revenues, linked to greater credibility with stakeholders. An increase in corporate sustainability standards positively affects complementary aspects of economic performance, particularly through reputation effects and through increased differentiation and innovation [44]. The regression model’s findings indicate that the EBITDA margin is unaffected by environmental performance. The company’s main focus in terms of environmental performance is empowering the community, rather than addressing the environmental impact caused by its operations. However, this impact is deemed insignificant and does not have a significant effect on the company’s financial performance [120]. Instead, the governance pillar has a positive effect on EBITDA margin. These results are in line with [121], who found a positive relationship. They state that companies with good governance openly share information with the public, as it demonstrates management’s competence in managing the company. As can be seen from the Table 3, digitalization has a positive and significant impact on EBITDA margin and Revenues. Highly digitized companies can produce products with zero marginal distribution and production costs, thus being able to achieve huge revenues [122,123] found that investment in digitalization is profitable since it can lead to a significant increase in a company’s FP, as digitalization can significantly improve productivity and sales, but without leading to an acceleration of total costs. The results obtained in this context suggest that digitalization creates a strategic competitive advantage that is reflected in the optimization of the production process and the improvement of company performance through increased production efficiency [124].

Table 3 presents the moderation effect of Digitalization (SDG 9) in the relation between the three ESG pillars and FP. The regression results show that the interaction terms, namely, Epillar_SDG9 and Gpillar_SDG9, have a significantly negative moderating impact on the EBITDA MARGIN and REVENUES, respectively. Since the sample analyzed covered the period between 2019 and 2021, this result could be explained by the early stage of the digitalization process, that did not unfold all of it long-term effects, so far. So, we have not been able to see the benefits of investments in digitalization, yet, but only the efforts in terms of investment costs.

During the period we have analyzed, the COVID-19 pandemic has greatly accelerated the digitalization of services. Numerous industries have experienced significant disruptions, leading many sectors to completely restructure their workflows, processes, and procedures [125]. In this context, digitalization has not been a strategic investment, but survival operational response to face the emergency. This may have caused inefficiencies that are reflected in financial efficiency indicators. Hence, investments in digitalization did not increase the value created by the company, as they only served to substitute the way some support activities were carried out before the COVID-19 outbreak.

Furthermore, this explanation is paradoxically coherent with the contradicting findings that succeeded in proving the moderating role of digitalization between ESG and firm performance [2]. In fact, the results of the previous study [2] are obtained by investigating mostly different variables (e.g., Tobin’s Q, cash flow, debt ratio, single ESG pillars: E, S, G) as well as under different conditions of analysis compared with our study. First, the two works utilize different data sources: firms listed in the Indonesian Stock Exchange vs our Refinitiv Eikon dataset–- that we selected (also) because it is specialized in the provision of ESG scores. Second, the research efforts are conducted within different macro- and micro-economic contexts: only Indonesian firms with single-country data relying on a developing economy context vs firms included in Refinitiv Eikon from countries of the American/European/Oceanian continents (i.e., mostly developed economies). Thus, our study ensures a wider (multi-country) geographical and macro-economic coverage. Third, our work weighs less the pre-pandemic period as we focus on 2019–2021, while the previous study [2] on 2017–2021, whereas 60% of their firm-year data refer to an obsolete time by now. This further enforces our explanation that the recent investments in digitalization have not shown all their effects, yet, and more time is needed to capture their impact. Fourth, variables are partly different either in terms of conceptualization or operationalization. Fifth, the granularity of the statistical analysis conducted for the ESG scores is higher in our study, as we do not only consider the overall ESG score, but also the single score for ach pillar: E scores, S scores, G scores. Sixth, we make a specific sectoral choice for our analysis (i.e., energy and utilities sectors), thus, our results offer higher reliability and direct applicability to those sectors, as we do not investigate simultaneously firms from multiple industries/sectors.

## 5. Conclusions and implications

This study examines the effect of ESG on FP and the moderating role of digitalization in this relationship by analyzing 303 listed companies in the energy and utilities sector for 2019 and 2021, using data from Refinitiv Eikon. The regression models show that ESG positively affects firm revenue because customers reward good ESG strategies, boosting short-run performance. ESG strategies can add value to a company’s products and boost buyers and investors’ desire to buy, resulting in increased business revenue. In addition, the findings showed that the relationship between ESG and FP is not unique and positively correlated on each of the three ESG pillars. These results are coherent with a stream of the field literature that demonstrates the existence of a significant relationship among ESG and FP [61,62]. Beyond this, our findings suggest a positive relationship that confirms the general tendency of previous studies [9–11,60], whilst disowns the existence of a negative impact reported in other studies [5,13–18]. In addition, each ESG pillar plays a different role in the intertwined dynamics involving SDG9, FP and ESG, thus, depicting a more complex scenario deserving further in-depth analyses on single ESG pillars [26].

However, our findings present even more contrasting results if compared to other studies that do not find any significant relationship among the investigated variables [23,38,63–65].

Our second objective was to analyze the moderating influence of digitalization on the ESG-FP relationship. The findings indicate that digitalization, measured by SDG 9, when combined with ESG pillars, in particular E and G pillars, adversely affects a firm’s FP (REVENUES and EBITDA MARGIN). However, digitalization analyzed individually has a significant and positive impact on the same FP indicators.

This result, however, can be affected by the period analyzed, since only the early stage of the digitalization process accelerated by the COVID-19 outbreak was considered, whereas the investments costs of E and G pillars were not covered by the expected long-term benefits, yet. Such a possible explanation is further supported by the fact that the S pillar has not shown any significant impact, yet.

In addition, only one study [2] investigated the moderating role of digitalization, succeeding in proving its significant positive impact on the ESG-FP relationship. This result is not confirmed by our research, that proves a significant, but negative role of digitalization as a moderator, whilst we also prove a direct, significant positive effect on FP.

Therefore, future research should investigate if these findings are confirmed in the mature stage of the digitalization process. Beyond this limitation related to the time, this study suffers from other limitations which can direct future research. First, we focused on Energy and Utilities-sector firms only. The study can be extended in the future to cover other sectors. In this study, the short-term financial effect of digitalization was examined, but the results do not reflect its long-term mechanism. Theoretically, digitalization will have a sustainable effect on FP, so future research can focus more on the long-term effects of digitalization. Another limitation of our study may be in the variable used to represent digitalization. Future research could use a different indicator of digitization. In addition, since moderation changed the results significantly, future research should test additional variables and more complex scenarios of analysis, as suggested also by [22].

This pioneering study paves the way of a better understanding of the relationship between ESG and FP, and the impact of digitalization as a moderator. Moreover, the results of this study may have diverse practical implications. For managers, the results of this study suggest that ESG integration in corporate organizations positively affects FP because a strong ESG proposition enables businesses to grow both in existing and new markets. Therefore, managers should be confident that customers tend to reward ESG investments and to perceive the increased value of a company that poses ESG approaches at the forefront of its managerial strategies. Hence, the initial investments done in order to implement ESG initiatives can be undertaken by managers, as long-term effects of ESG promise to be beneficial to firms. However, the practical implementation of ESG investments suggests that executives and managers should allocate resources to ESG activities through adopting more efficient and rigorous approaches. This is very important when considering that the impact of ESG activities only unfold their entire magnitude after some years. Then, efficiency plays a key role in making the ESG investments affordable and sustainable during their initial phase. Additionally, the study’s findings support the investment in digitization by demonstrating notable enhancements in business operations, including higher financial efficiency, which could eventually strengthen company revenues. However, managers should consider that the beneficial effects of digitalization are not captured in full by our analysis, since we have limited the time up to 2021, whilst the recent large investments in digitalization occurred during the pandemic and have to show their entire impact, yet. For instance, all the National Recovery and Resilience Plans throughout the European Union started after 2021 and are still in operation. Thus, this pragmatically explains why the digitalization efforts show a negative impact and are not mature enough to do a U-turn and prove to be beneficial. However, as for [26] research, managers should support the creation of as well as nurture digital transformation capabilities at the organizational level to improve corporate financial performance.

Under the theoretical perspective, this work builds on the adoption of the IST framework [2] and represents a novel application of this theoretical framework in literature. However, we conceptualized the theoretical framework with an in-depth separation of the three ESG pillars to obtain a more nuanced theoretical understanding of the cross section of the ESG roles in connection with digitalization and corporate financial performance.
